# Ergonomic risk assessment of smartphone users using the Rapid Upper Limb Assessment (RULA) tool

**DOI:** 10.1371/journal.pone.0203394

**Published:** 2018-08-30

**Authors:** Suwalee Namwongsa, Rungthip Puntumetakul, Manida Swangnetr Neubert, Sunisa Chaiklieng, Rose Boucaut

**Affiliations:** 1 Research Center in Back, Neck, Other Joint Pain and Human Performance (BNOJPH), Khon Kaen University, Khon Kaen, Thailand; 2 School of Physical Therapy, Faculty of Associated Medical Sciences, Khon Kaen University, Khon Kaen, Thailand; 3 Program of Production Technology, Faculty of Technology, Khon Kaen University, Khon Kaen, Thailand; 4 Department of Environmental Health Science, Faculty of Public Health, Khon Kaen University, Khon Kaen, Thailand; 5 School of Health Sciences (Physiotherapy) University of South Australia, iCAHE (International Centre for Allied Health Evidence), Adelaide, Australia; University of Minnesota, UNITED STATES

## Abstract

The number of smartphone users globally is rapidly increasing. This study aimed to assess the level of ergonomic risk to smartphone users, and to evaluate the correlation between any self-reported musculoskeletal disorders and the level of ergonomic risk. Thirty participants completed a questionnaire, tailored specifically for smartphone users, to determine any musculoskeletal disorders. Participants were given a seated smartphone texting task and their postures were video recorded during the task. The video scenarios were evaluated by three independent researchers to determine the level of ergonomic risk using the Rapid Upper Limb Assessment (RULA) tool. RULA is an observation-based screening tool which has been widely used to assess the postural risk of IT device users. However, it has not yet been specifically utilized with smartphone users. The RULA tool scores identified ergonomics risks from using smartphones to text. Most smartphone users had a total RULA Grand Score of 6 for both sides (left side: 80.00%, right side: 90.00%), indicating the need for further investigation and changes (Action Level 3). Notably, no participants had acceptable RULA Grand Scores of 1 or 2. The correlation between musculoskeletal disorders and the ergonomic risk among smartphone users was determined using the Chi-Square test and Fisher's exact test; *p*<0.05 was considered statistically significant. There was a significant correlation between right RULA Grand Score and neck musculoskeletal disorder (χ2 = 9.424 at *p* value = 0.009) and right RULA Grand Score and upper back musculoskeletal disorder (χ2 = 31.717 at *p* value <0.001). RULA Score B (combination of neck, trunk and leg postures) and RULA Score D (combination of Score B, muscle use and force scores for group B) were also significantly correlated with neck musculoskeletal disorders (χ2 = 19.286 at *p* value<0.001 and χ2 = 9.310 at *p* value = 0.002 respectively). The RULA results identified the high ergonomics risk of smartphone users, this resulted from two key risk factors: posture and muscle use. The neck, trunk and leg postures had a combined effect on neck musculoskeletal disorders. Future investigations should consider these factors when designing ergonomic interventions for smartphone users.

## Introduction

In our digital society, the use of smartphones has increased rapidly. There are 3.4 billion smartphone users worldwide [1]. Thailand is nineteenth of the top twenty-five countries in terms of global smartphone use [2]. Smartphone users vary in age, ranging from students to workers to senior citizens [3]. Coincident with growing smartphone use, concerns of musculoskeletal problems associated with intensive smartphone use have also increased. An epidemiological study of smartphone users conducted in the Republic of Korea found that 18.8% of smartphone users experienced musculoskeletal symptoms in at least one body part [4], especially in the neck, upper trunk and upper extremity. In another Korean study involving smartphone users, Kim et al. (2015) found the most painful body region from smartphones use was reported to be the neck (55.8%) [5]. Similarly, in Thailand, Namwongsa et al. [6] demonstrated that neck pain was the most prevalent musculoskeletal disorder in smartphone users. Moreover, in the cross-sectional studies, neck and/or shoulder symptoms among mobile touch screen devices users were reported to have the highest prevalence rates, ranging from 26.3% to 60% [7].

Previous studies show that physical risk factors which are related to neck musculoskeletal disorders in workers include awkward postures [8–12]. Prolonged smartphone use can cause various musculoskeletal problems [13]. In particular, smartphone use can encourage awkward postures. A previous study in Thailand [6] found that the majority of smartphone users who reported musculoskeletal disorders adopted positions in the upper body of: neck flexion (82.74%), shoulder protraction (56.61%), elbow flexion (65.16%), wrist and hand flexion during keying (22.40%), and wrist and hand supination to support the device (21.62%). The investigators also reported upper back flexion (67.50%) and lower back flexion (43.23%) positions being adopted during smartphone use. These awkward postures can affect soft tissues (e.g. strain muscles and ligaments, irritate tendons, compress nerves) leading to musculoskeletal discomfort.

**Fig 1:**
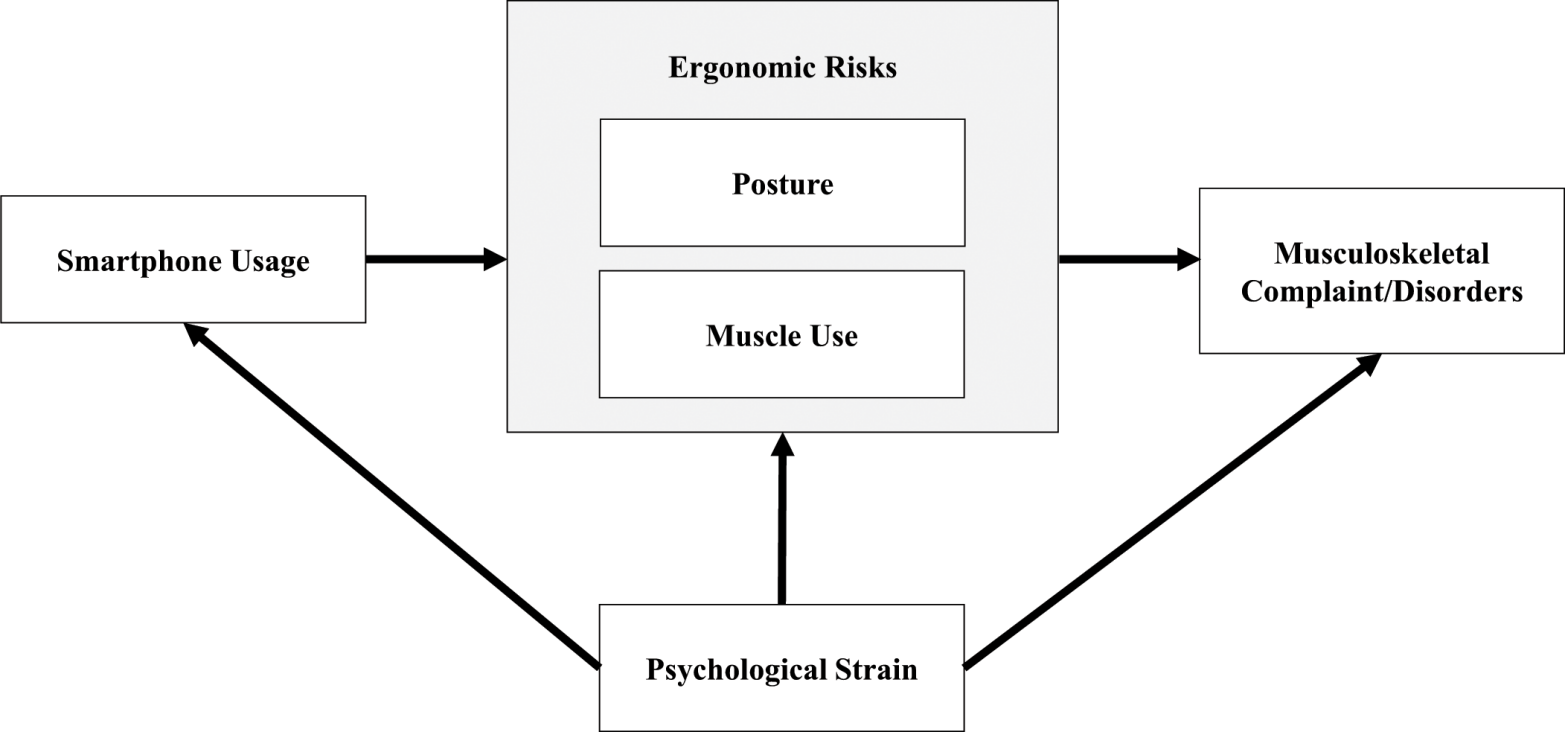
A conceptual model of smartphone usage, adapted from Dennerlein (2015).

A conceptual model in this study was adapted from the mobile computing technology model proposed by Dennerlein [14]. Smartphone usage may increase ergonomic risks of posture and muscle use, as well as psychological strain, which can lead to musculoskeletal complaints and disorders (Fig 1). Various methods have been reported to assess ergonomic risks when using IT devices including using the Rapid Upper Limb Assessment tool (RULA) [15–18] and a 3D Motion Analysis System [19–21]. The RULA tool, developed by Corlett and McAtamney (1993), is a screening tool based on observation, which is used to assess exposure to load factors due to posture of the neck, trunk and upper limb along with muscle use and forces (external loads). Administration of this inexpensive tool does not require special equipment or pre-existing skills [22]. Using the RULA tool comprises assigning a numerical rating to the posture of the upper arms, lower arms and wrists (Score A) together with posture of the neck, trunk and legs (Score B), and then assigning another numerical rating for additional factors that strain the musculoskeletal system, such as repetitive action, static loading and force exertion so they become Score C (Score A + muscle use + force scores for the group A) and Score D (Score B + muscle use + force scores for group B) respectively. These ratings are scored using an algorithm to compute a Grand Score ranging from 1 to 7, and an Action Level ranging from 1 to 4 that has associated implications for remedial action. The RULA tool allows the left and right upper limbs to be assessed separately, yielding a Grand Score and Action Level for each side of the body. Previous ergonomic studies have used the RULA tool to estimate the posture of children when conducting academic tasks at computer workstations in the classroom setting [23]. To date, no reported studies have used the RULA tool to implement ergonomic risk assessments on smartphone users. Currently, there are no risk assessment tools developed to evaluate the specific ergonomic hazards of smartphone use. While using a smart phone, static postures include head, neck, trunk, upper arms, lower arms, wrists and leg postures. These postures provide a stable base for some parts of body performing in repetitive manners (e.g., thumb or other fingers). Previous epidemiological studies reported smartphone users to have highest prevalence of neck musculoskeletal disorders, which was our body part of interest. In this case, RULA is an appropriate tool for assessment. Although absence of high force exertion for smartphone usage, David [24] recommended using RULA for upper body and limb assessment of such task.

Self-reported musculoskeletal disorders can be assessed by various questionnaires, the Standard Nordic Questionnaires (SNQ) is the common questionnaire to assess the prevalence of body part musculoskeletal discomfort within the last 7 days, last 12 months and trouble preventing normal work within last 12 months. The SNQ is used to survey musculoskeletal disorders in the neck, shoulder, elbow, wrist and hand, upper back, lower back, hip and thigh, knee, ankle and foot. All answers are on a dichotomous scale (yes or no). The validity of SNQ comparing the last 7 days and physical examination is 0.8. The reliability and sensitivity of SNQ within the last 7 days and the last 12 months is 0.66–0.92 and 0.74–0.93, respectively [25]. The reliability of the Thai version was reported to be 0.66–1 [26]. Therefore, the SNQ comprises high validity, good test-retest reliability and sensitivity in the measurement of the prevalence of musculoskeletal disorders [27].

Previous studies have yet not assessed ergonomic risk levels and correlations between musculoskeletal disorders and ergonomic risk levels among smartphone users. Therefore, our study aimed to assess ergonomic risk levels exposed by smartphone users using the RULA tool. The objective of the study was also to determine any correlations between musculoskeletal disorders and ergonomic risk levels. We hypothesized that RULA scores, which indicating ergonomic risk levels, has correlations with the percentage of musculoskeletal disorders, which was assessed by SNQ. Specially, we expected neck posture wound correlated with percentage of neck musculoskeletal disorders based on results of our previous study [6] indicating neck flexion posture was a factor associated with neck musculoskeletal disorders. Thus, the result of our study could inform ergonomic guidelines concerning the use of smartphones, which should be develop interventions to reduce the risk of musculoskeletal disorders.

## Materials and methods

### Study design

The study design was cross-sectional in nature. The research proposal for this study was submitted to and approved by, the Khon Kaen University Ethics Committee in Human Research before beginning the study (HE591321).

### Sample size

A sample size estimation was conducted as described by Hulley et al. (2013) [28]. A power calculation was conducted based on a critical α-value of 0.05 and a 1-β of 0.2. To reach a power level of 80%, 30 participants were required.

### Participants

Participants were recruited for this study by printed media advertisement posted on notice boards, at the Khon Kaen University in Thailand. The 30 participants were smartphone users, selected using a purposive sampling method. The inclusion criteria were: 1) young adult aged between 18–25 years, 2) owner of an iPhone 5s smartphone, 3) at least six month’s experience in using smartphones and, 4) daily smartphone use of at least 2 hours per day.

Participants were excluded from the study if they had any of the following conditions: 1) any history of traumatic injuries or surgical interventions of relevant regions within the past year such as whiplash injury, 2) other medical conditions which may have a negative effect on the spine and upper extremities such as deformity, 3) chronic diseases affecting the musculoskeletal system such as rheumatoid arthritis, osteoarthritis and other connective tissue disorders (such as fibromyalgia), 4) neurological and orthopedic disorders as well as sensory deficit, 5) visual problems (not corrected by glasses), dizziness or vertigo and, 6) consumption of any sedative drug or alcohol within the past 48 hours.

All participants who met the inclusion criteria were invited to participate in this study. Written informed consent was provided to each participant prior to the study.

### Procedures

The procedures are described in five discrete steps. First, each participant was asked to complete the musculoskeletal disorders among smartphone users questionnaire. This questionnaire consists of five sections: (1) demographic data, (2) smartphone use data, (3) use of other devices data, (4) the Suanprung Stress Test-20, and (5) a modified Thai version of Standardized Nordic Questionnaire (SNQ) [26]. The reliability and validity of the modified Thai version of SNQ were established in a pilot study (accepted as a high validity tool; validity (IOC) = 0.6–1 and accepted as a moderate reliability tool; reliability (Cronbach Alpha = 0.71) [29]. Second, each participant was then asked to use their smartphone while a video recording was taken. Third, the three investigators viewed each participant’s entire video clip together and reached a consensus on which part of the video showed the most sustained posture. Fourth, the three investigators independently used the RULA tool to assess this sustained posture and determine a Grand Score for each side of the body, thus one RULA assessment for the left side and one for the right. Fifth, the correlation between musculoskeletal disorders and ergonomic risk among smartphone users was conducted using statistical analysis.

### RULA training and researcher reliability rating

The data analysis was conducted by three independent researchers, each with many years of experience in the ergonomics field. They all attended a one-hour training session on the use of RULA given by an expert ergonomist; then all of the researchers participated in intra-rater reliability ICC (3,1) (0.926–0.976) and inter-rater reliability (0.922–0.951) tests before starting the study.

### Video scenarios–collecting the data

The 30 participants were asked to text responses to one of the researchers on their smartphone (using the most popular texting application in Thailand, Line application by NHN Japan Inc.). They sat on general lecture chair for 10 minutes to do this [30–33]. The participants were instructed to text at their customary speed and as accurately as possible, without having to amend any error while texting or use the automatic “word complete” function during texting or use the sticker or emoji instead of their words. Prior to the actual data collection, the participants were given 3 minutes to become familiar with texting [34] in the laboratory environment. Three cameras were used to record the participants engaged in smartphone use in the anterior (front) and lateral views (right and left sides). The cameras were set at a distance that allowed for clear views of the participants using their smartphones. The point of setting the cameras and chair position (to use a smartphone) was to have the participants sit in the same location throughout the video recordings.

### Video scenarios–reviewing and analyzing the data

The three researchers together viewed each of the participant videos which were adjusted to slow the speed of movement for more precise and accurate analysis [35]. The most sustained posture of smartphone users during texting for 10 minutes was identified and the level of ergonomic risk was analyzed independently by the three researchers. The level of ergonomic risk was identified according to RULA Grand Score categories: “1 or 2 indicates that the posture is acceptable if not maintained or repeated for long periods. 3 or 4 indicates that further investigation is needed, and changes may be required. 5 or 6 indicates that changes will be required soon. 7 indicates that changes are required immediately” [22].

### Statistical analysis

Descriptive statistics were used to analyze characteristics of participants and musculoskeletal disorder variables. Continuous variables, including age, weight, height, study hours per day, years of smartphone usage, average smartphone usage hours per occurrence/day/year, years of using other devices, average usage of other devices in hours per occurrence/day/year, were analyzed by mean and standard deviation (SD). Categorical variables, including sex, BMI, hand dominance, smoking behavior, drinking behavior, exercise behavior, underlying disease, underlying musculoskeletal disease, accident history, the use of smartphones data, stress level, musculoskeletal disorders and level and ergonomic risks were considered in terms of frequency and percentage. The Chi-Square test and Fisher's exact test were used to quantify the relationship between the musculoskeletal disorders and the ergonomic risk among smartphone users. The variables with p-value less than 0.05 were considered statistically significant. Data were analyzed using the STATA program version 10 (STATA, College Station, TX, USA).

## Results

### General characteristics of participants

The general characteristics of smartphone user participants in this study are presented in Table 1. There were 4 male (13.30%) and 26 female (86.70%) smartphone users. The majority of participants reported: a BMI value in the normal range (100.00%), all had right hand dominance (100%), no history of smoking (93.30%) or never drinking alcohol (56.70%), currently engage in exercise (56.70%), no underlying disease (86.70%), no underlying musculoskeletal disease (100%) and no accident history (93.30%). The average age of participants was 21.43±1.40 years, weight was 52.50±9.26 kilograms, height was 161.76±8.23 centimeters and study hours per day was 6.20±1.97. Over half (53.30%) of the smartphone users reported high stress.

**Table 1:** General characteristics of the smartphone users (n = 30).

Characteristics	n (%)	Mean±SD	Min-Max
**Sex**			
**Male**	4 (13.30)		
**Female**	**26 (86.70)**		
**Age (years)**		21.43±1.40	18.00–25.00
**Weight (kilograms)**		52.50±9.26	40.00–80.00
**Height (centimeters)**		161.76±8.23	148.00–188.00
**Body Mass Index (kg/m^2^)**			
**Normal**	**30 (100.00)**		
**Overweight (≥25 kg/m^2^)**	-		
**Study hours per day (hour/day)**		6.20±1.97	1.00–10.00
**Hand dominance**			
**Right**	**30 (100.00)**		
**Left**	-		
**Both**	-		
**Smoking behavior**			
**Current smoker**	-		
**Former smoker**	2 (6.70)		
**Never smoker**	**28 (93.30)**		
**Alcohol drinking behavior**			
**Current drinker**	2 (6.70)		
**Former drinker**	11 (36.70)		
**Never drinker**	**17 (56.70)**		
**Exercise behavior**			
**Currently exercise**	**17 (56.70)**		
**Formerly exercised**	5 (16.70)		
**Never exercised**	8 (26.70)		
**Underlying disease**			
**Yes**	4 (13.30)		
**No**	**26 (86.70)**		
**Musculoskeletal underlying disease**			
**Yes**	-		
**No**	**30 (100.00)**		
**Accident history**			
**Yes**	2 (6.70)		
**No**	**28 (93.30)**		
**Suanprung stress test level**			
**Mild stress**	5 (16.70)		
**Moderate stress**	7 (23.30)		
**High stress**	**16 (53.30)**		
**Severe stress**	2 (6.70)		

### Characteristics of participant smartphone use

The characteristics of participant smartphone use were presented in Table 2. Respondents reported they had used smartphones for 5.36±2.48 years, with duration of smartphone use 1.16±1.36 hours per time for 6.73±3.12 hours per day. Additionally, more than half the participants used smartphones in the evening (90.00%) and they had rest time during use (83.30%). Participants mainly used their right hand (73.30%), and reported they held their smartphone in both hands and entered text using both thumbs (50.00%). Participants reported mainly texting in a sitting posture (73.30%) for social networking purposes such as Facebook and LINE application (86.70%). In addition, participants also reported using other devices (90.00%) especially laptop (63.30%) which they reported they had used for the previous 5.08±3.52 years, used 1.81±1.21 hours per time and used 3.48±1.78 hours per day.

**Table 2:** Characteristics of the smartphones used by participants (n = 30).

Characteristics	n (%)	Mean±SD	Min-Max
**The start time until to current time (years)**		5.36±2.48	1.00–11.00
**Using time per time (hours)**		1.16±1.36	0.20–8.00
**Using time per day (hours)**		6.73±3.12	3.00–15.00
**Time period**			
**Evening**	**27 (90.00)**		
**Others (such as before bed)**	3 (10.00)		
**Rest time**			
**Yes**	**25 (83.30)**		
**No**	5 (16.70)		
**Main hand**			
**Only right side**	**22 (73.30)**		
**Only left side**	-		
**Both sides**	8 (26.70)		
**Data entry method**			
**Hold in both hands and data entry by both thumbs**	**15 (50.00)**		
**Hold by both hands and data entry by right thumb**	5 (16.70)		
**Hold by right hand and data entry by right thumb**	10 (33.30)		
**Posture during use**			
**Sitting**	**22 (73.30)**		
**Lying**	8 (26.70)		
**Purpose of use**			
**Social network (such as Facebook, Line application)**	**26 (86.70)**		
**Entertainment (such as TV, Clip, Movies and radio)**	2 (6.70)		
**Others (such as game playing)**	2 (6.70)		
**Other device use**			
**Yes**	**27 (90.00)**		
**No**	3 (10.00)		
**Other device types**			
**Laptop**	**19 (63.30)**		
**Personal computer**	4 (13.30)		
**Tablet**	4 (13.30)		
**Did not use**	3 (10.00)		
**From start time to current time, use of other devices (years)**		5.08±3.52	0.50–12.00
**Use time per occasion of other devices (hours)**		1.81±1.21	0.20–4.00
**Use time per day of other devices (hours)**		3.48±1.78	1.00–8.00

### Musculoskeletal disorder of the smartphone users

Results of SQN showed that the musculoskeletal disorders was highest in the neck (90.00%), followed by shoulder 73.30%, upper back 63.30%, wrist and hand 36.70% and lower back 30.00%. Musculoskeletal disorders were less prevalent in the hip and thigh 13.30%, the knee 13.30%, the ankle and foot 10.00% and the elbow 6.70%.

### Ergonomic risk using RULA

The RULA scores of the smartphone users are shown in Table 3. The mean upper arm posture scores of smartphone users were 1.27±0.450 (left side) and 1.37±0.490 (right side), while the average lower arms posture scores were 1.97±0.183 for both sides. The average wrist posture scores were 3.03±0.890 for left side and 3.27±0.691 for right side, while the average wrist twist posture scores were 1.20±0.407 for left side and 1.10±0.305 for right side. The mean neck, trunk and legs posture scores were 3.73±0.691, 3.30±0.988, 1.70±0.466 respectively. The mean upper and lower extremities muscle use scores were 1±0.00 and 1±0.00 respectively. The mean upper extremities forces scores were 0.23±0.626 for left side and 1±0.00 for right side and mean lower extremities force sub score was1.60±0.621.

**Table 3:** The RULA scores of the smartphone users.

Body part, n (%)																									
RULA score	Upper Arms Posture		Lower Arms Posture		Wrists Posture		Wrists Twist Posture		Score A (Upper arms, lower arms and wrists Postures)		Muscle Use for Group A		Force for Group A		Score C (Score A + muscle use and force scores for group A)		Neck	Trunk	Legs	Score B (Neck, Trunk, Leg Postures)	Muscle Use for Group B	Force for Group B (Highest score = 3)	Score D (Score B + muscle use and force scores for group B)	Grand Score	
	(Highest score = 6)		(Highest score = 3)		(Highest score = 4)		(Highest score = 2)				(Highest score = 1)		(Highest score = 3)				(Highest score = 6)	(Highest score = 6)	(Highest score = 2)		(Highest score = 1)			(Highest score = 7)	
	Lt.	Rt.	Lt.	Rt.	Lt.	Rt.	Lt.	Rt.	Lt.	Rt.	Lt.	Rt.	Lt.	Rt.	Lt.	Rt.								Lt.	Rt.
**0**													**26**	**30**								2			
													**(86.70)**	**(100.00)**								(6.70)			
**1**	**22**	**19**	1	1	2		**24**	**27**			**30**	**30**						2	9		**30**			0	0
	**(73.30)**	**(63.30)**	(3.30)	(3.30)	(6.70)		**(80.00)**	**(90.00)**			**(100.00)**	**(100.00)**						(6.70)	(30.00)		**(100.00)**				
**2**	8	11	**29**	**29**	5	4	6	3	5	2			4				1	4	**21**			**28**		0	0
	(26.70)	(36.70)	**(96.70)**	**(96.70)**	(16.70)	(13.30)	(20.00)	(10.00)	(16.70)	(6.70)			(13.30)				(3.30)	(13.30)	**(70.00)**			**(93.30)**			
**3**					**13**	**14**			**22**	**26**					1	5	9	8		1				0	0
					**(43.30)**	**(46.70)**			**(73.30)**	**(86.70)**					(3.30)	(16.70)	(30.00)	(26.70)		(3.30)					
**4**					10	12			3	2					**23**	**22**	**17**	**15**		1			1	0	1
					(33.30)	(40.00)			(10.00)	(6.70)					**(76.7)**	**(73.3)**	**(56.70)**	**(50.00)**		(3.30)			(3.30)		(3.30)
**5**															4	3	3	1		6				0	0
															(13.30)	(10.00)	(10.00)	(3.30)		(20.00)					
**6**															2					8			1	**24**	**27**
															(6.70)					(26.70)			(3.30)	**(80.00)**	**(90.00)**
**7**																				**11**			3	6	2
																				**(36.70)**			(10.00)	(20.00)	(6.70)
**8**																				3			6		
																				(10.00)			(20.00)		
**9**																							**8**		
																							**(26.70)**		
**10**																							**8**		
																							**(26.70)**		
**11**																							3		
																							(10.00)		
**Mean**	1.27	1.37	1.97	1.97	3.03	3.27	1.20	1.10	2.93	3.00	1	1	0.27	1	4.23	3.93	3.73	3.30	1.70	6.20	1	1.87	8.80	6.20	6.00
**(SD)**	(0.450)	(0.490)	(0.183)	(0.183)	(0.890)	(0.691)	(0.407)	(0.305)	(0.521)	(0.371)	(0.000)	(0.000)	(0.691)	(0.000)	(0.626)	(0.521)	(0.691)	(0.988)	(0.466)	(1.186)	(0.000)	(0.507)	(1.562)	(0.407)	(0.455)

The final RULA Grand Score of smartphone users ranged from 6 (n = 24, 80%) to 7 (n = 6, 20%) with an average RULA Grand of 6.20±0.407 for the left side. For the right side, the RULA Grand Score included: minimum score of 4 (n = 1, 3.30%), mode score of 6 (n = 27, 90.00%), and maximum score of 7 (n = 2, 6.70%), with an average RULA Grand of 6.00±0.455. Most smartphone users had a total Grand Score of 6 for both sides, which the RULA tool indicates means they require further investigation and changes soon (Action Level 3). It was notable that no participants had acceptable RULA scores (which would have scores ranging from 1–2).

### Correlation between musculoskeletal disorders and level of ergonomic risk among smartphone users

The correlation between musculoskeletal disorders and level of ergonomic risk among smartphone users is presented in Table 4. This study found significant correlations between neck musculoskeletal disorder and right RULA Grand Score (χ2 = 9.424 at *p* value = 0.009). Upper back musculoskeletal disorder and right RULA Grand Score (χ2 = 31.717 at *p* value < 0.001) were also significantly correlated. Additionally, RULA Score B (combination of neck, trunk and leg postures) had significant correlation with neck musculoskeletal disorders (χ2 = 19.286 at *p* value < 0.001). RULA Score D (combination of Score B, muscle use and force scores for group B) had significant correlation with neck musculoskeletal disorders (χ2 = 9.310 at *p* value = 0.002).

**Table 4:** Correlation between musculoskeletal disorders and level of ergonomic risk among smartphone users (n = 30).

RULA	Musculoskeletal disorders	Chi-square (*p* value)	
		Lt.	Rt.
Upper arms posture	Shoulder	2.456 (0.483)	0.851 (0.837)
Lower arms posture	Elbow	0.074 (0.964)	0.074 (0.964)
Wrists posture	Wrist and hand	3.325 (0.344)	0.720 (0.868)
Wrist twist posture	Wrist and hand	1.115 (0.773)	6.720 (0.081)
Score A (Upper arms, lower arms and wrists posture)	Shoulder	1.327 (0.723)	2.618 (0.454)
	Elbow	0.153 (0.926)	0.429 (0.807)
	Wrist and hand	1.241 (0.743)	2.463 (0.482)
Neck posture	Neck	1.667 (0.197)	
Trunk posture	Upper back	9.614 (0.222)	
	Lower back	2.066 (0.151)	
Leg posture	Hip and thigh	1.978 (0.160)	
	Knee	1.978 (0.160)	
	Ankle and foot	1.429 (0.232)	
Score B (Neck, Trunk, Leg posture)	Neck	19.286 (<0.001)**	
	Upper back	3.701 (0.296)	
	Lower back	0.408 (0.523)	
	Hip and thigh	0.330 (0.566)	
	Knee	0.330 (0.566)	
	Ankle and foot	0.238 (0.626)	
Score C (Score A+muscle use and force for group A)	Shoulder	1.787 (0.618)	2.618 (0.454)
	Elbow	0.074 (0.964)	0.429 (0.807)
	Wrist and hand	0.599 (0.897)	2.463 (0.482)
Score D (Score B+muscle use and force for group B)	Neck	9.310 (0.002)*	
	Upper back	1.787 (0.618)	
	Lower back	2.414 (0.120)	
	Hip and thigh	0.159 (0.690)	
	Knee	0.159 (0.690)	
	Ankle and foot	0.115 (0.735)	
Grand Score	Neck	0.370 (0.543)	9.424 (0.009)*
	Shoulder	2.585 (0.460)	4.312 (0.635)
	Elbow	0.536 (0.765)	0.238 (0.993)
	Wrist and hand	1.335 (0.721)	1.930 (0.926)
	Upper back	5.426 (0.143)	31.717 ((<0.001)**
	Lower back	0.040 (0.842)	3.192 (0.203)
	Hip and thigh	2.596 (0.107)	0.513 (0.774)
	Knee	0.072 (0.788)	0.513 (0.774)
	Ankle and foot	0.833 (0.361)	0.370 (0.831)

*p<0.05

**p<0.001

## Discussion

In this study, the level of ergonomic risk among smartphone users was assessed using the RULA tool. No participants had acceptable RULA scores (Grand Score of 1 or 2). Most smartphone users had high levels of ergonomics risk, a Grand Score of 6 on both sides (left side: 80.00%; right side: 90.00%), which requires investigation and changes soon (Action Level 3). There was a significant correlation between the right RULA Grand Score and musculoskeletal disorders of the neck and upper back. RULA Score B and RULA Score D also had significant correlation with neck musculoskeletal disorders.

We now consider the possible reasons why smartphone users had high ergonomic risk when they were using their smartphone. There are three components of RULA assessment, which related to these risk levels including posture, muscle use and force scores [22]. Regarding the posture score, observation of the posture of the body part revealed that while using the smartphone most participants held both their upper arms in flexed postures between -20 to +20 degrees, but some participants also raised their shoulder or leaned or supported their upper arm too. Both lower arm postures were in flexion positions of more than 100 degrees while their wrist postures in both flexion and extension positions were between -15 to +15 degrees with their wrists bent away from the midline while the wrist twist postures were mainly in the hand-shake position (mid-range of twist). Comfortable working posture requires an arm angle of less than 20 degrees in both the sagittal and frontal planes [36].

Participants also held their neck in more than 20 degrees flexion together with neck twisted or side bent. Flexed postures, such as these, are well-known causes of neck pain [37]. A small forward movement of the head in the sagittal plane increases the load on the supporting structures and stimulates the cervical muscles [38]. Harrison et al. (1999) found that the compressive load on the cervical discs in the neck-forward flexed position was 10 kilograms greater than that in the upright neck position [39]. The risk of neck pain increases when the neck is rotated more than 45 degrees for more than 25% of the work time. An increase in risk also occurs when the neck is flexed more than 45 degrees from the natural neck position for more than 5% of working time. However, even if the neck is flexed at 20 degrees for more than 40% of work time, the risk increases rapidly with time [40].

A trunk flexion posture of participants between 20 to 60 degrees was demonstrated while some also had their trunk twisted or side bent. Bending the trunk forward/backward may be classified in terms of one of four load zones. The zone relating to optimal working posture, refers to bending up to 20 degrees, the second from 20 degrees to 60 degrees. When the trunk is bent forward more than 60 degrees or when the trunk is bent backwards the risk of developing musculoskeletal disorders increases rapidly. For the trunk, bending sideways (frontal plane) or twisting the upper part with respect to lower part (transverse plane) determines the comfort zones with a criterion of 10 degrees. Awkward trunk posture (twisting or bending) is also a strong risk factor in absenteeism due to back pain [36]. Moreover, the posture of the neck, trunk or shoulders may be effected by the location of the user's hands and where they are looking.

Participants’ leg posture was not well supported or evenly balanced. Sometimes at the workplace the legs can also be exposed to awkward positions. For example when used, a foot pedal should be located at ground level in such a way as to avoid uncomfortable foot and leg positions [36].

These postures correspond with survey results from our previous study which found that smartphone users which found that smartphone users held their neck flexion, shoulder protraction, elbow flexion, wrist and hand flexion during keying, with their wrist and hand supination to support the device while their upper and lower back flexed and they also held hip and thigh flexion, knee flexion and ankle and foot neutral [41]. It was clear that each of these postures adopted by smartphone users is awkward [42]. The further a joint moves towards either end of its range of motion, or the further away from the neutral posture, the more awkward or poor the posture becomes, the more strain is put on the muscle, tendon and ligaments around the joint, and this can also compress nerves and irritate tendons [43–44]. Awkward postures such as arm raising, head and neck flexion, and forward bending of the trunk can lead to ergonomic problems and affect the level of ergonomics risk [45], so this must be addressed to prevent subsequent discomfort from musculoskeletal disorders.

With respect to the muscle use score, the smartphone user’s postures are mainly static; participants held their smartphone for longer than one minute or repetitively used their smartphone (actions repeated more than 4 times per minute can increase the RULA score (1 score) [22]. The questionnaire results indicate participants used their smartphone for 1.04±1.47 hours per time and normally used it 5.03±3.37 hours per day. Over three-quarters of the participants (76.70%) also reported that they had rest times during usage. Previous observational studies show smartphone users used their muscles mainly in a static manner [46] and for prolonged duration [42] which would affect their ergonomic risk levels.

Finally, regarding the forces score. The average weights of participants’ smartphones was 112 grams (plus 16.37+9.85 grams of average smartphone protector case weight) which is less than the 4.4 pounds or 2 kilograms a cut of score in RULA. Thus, the right upper limb forces score were considered to score 0 using the RULA tool [22]. Four participants (13.30%) held their smartphone in a static manner. They used their left upper limb actively to text and held their right upper limb statically, while 28 participants (93.30%) held their other lower limbs statically, so the force scores or these participants were increased (2 scores).

From the reasons stated above, it is clear that the high levels of ergonomics risk in smartphone users in this study were mainly affected by their posture and muscle use during smartphone usage. Significant correlations were found between neck and upper back musculoskeletal disorders with the right RULA Grand Score. This result can possibly be explained by considering that these two scores (posture and muscle use) in combination are the main factors that affect the correlation. In agreement with our conceptual model, smartphone usage may increase ergonomic risks of posture and muscle use, which can lead to musculoskeletal complaints and disorders. No significant correlations were found between neck and upper back musculoskeletal disorders with the left RULA Grand Score, this may be due to the left having less variation of Grand Scores than the right, and perhaps also because all participants were right handed.

Surprisingly, no significant correlation was found between neck posture and neck musculoskeletal disorders. However, neck musculoskeletal disorders had significant correlations with RULA Score B (combination of neck, trunk and leg postures) and RULA Score D (combination of Score B, muscle use and force scores for group B). It is clear that the neck, trunk and leg postures had a combined effect on neck musculoskeletal disorders. These postures consisted of 1) holding neck in more than twenty degree flexion or extending neck together with neck twisted or side bent, while 2) holding trunk flexion posture more than twenty degrees and twisted or side bent and 3) leg was not well supported or evenly balanced. Smartphone users should avoid any combination of these postures to prevent neck musculoskeletal disorders.

This study is the first study to utilize an observation-based screening tool to demonstrate the ergonomic risk level in smartphone users. The assessment of an individual’s exposure to ergonomic factors using the RULA tool can be conducted quickly and in real time during smartphone usage. The results showed that neck pain was the musculoskeletal disorder with the highest prevalence in smartphone users corresponding with findings of previous studies [6–7, 47]; further, that smartphone users had high ergonomic risk levels which were mainly the result of posture and muscle use. A limitation of this study is that we did not focus on repetitive parts of body although the experimental task was texting, which included repetitive finger motions. However, the repetitive manners should be further assessed by other specific tools such as Occupational Repetitive Action (OCRA). Besides the observation-based assessment tools, future studies would be interesting to use direct measurement methods, such as surface Electromyography (sEMG), to investigate muscle use for each posture in the neck region. Another limitation of the current study is that all participants were right handed and the sample was mainly comprised of females. In the future, it may be beneficial to have a more even gender mix of participants to compare results.

## Conclusion

Smartphone users in the current study adopted awkward postures, and they all had high ergonomic risk levels when using their smartphones. There was a significant correlation between the right RULA Grand Score and musculoskeletal disorders of the neck and upper back. While RULA Score B (combination of neck, trunk and leg postures) and RULA Score D (combination of Score B, muscle use and force scores for group B) also had significant correlation with neck musculoskeletal disorders. This study may provide useful information to practitioners who treat patients with neck pain who are smartphone users. Educational interventions which address the factors of posture (especially neck, trunk and leg postures) and muscle use may prove helpful in prevention or treatment of neck musculoskeletal disorders in smartphone users.

## Supporting information

S1 Table The Rapid Upper Limp Assessment (RULA) scores of university student smartphone users. (DOCX)

S2 Table The musculoskeletal disorders of university student smartphone users. (DOCX)
