# Supplementary material for: Ergonomic risk assessment of smartphone users using the Rapid Upper Limb Assessment (RULA) tool
Source: PLoS One. 2018 Aug 30;13(8):e0203394. doi: 10.1371/journal.pone.0203394 (PMC6117073; doi:10.1371/journal.pone.0203394)
Supplement: S1 Table — (DOCX) [file pone.0203394.s001.docx]

**S1 Table. The Rapid Upper Limp Assessment (RULA) scores of university student smartphone users.**

| ID | Upper Arms Posture  (Highest score=6) | | Lower Arms  Posture  (Highest score=3) | | Wrists  Posture  (Highest score=4) | | Wrists Twist  Posture  (Highest score=2) | | Score A  (Upper arms, lower arms and wrists Postures) | | Muscle Use  for Group A  (Highest score=1) | | | Force  for Group A  (Highest score=3) | | | Score C  (Score A+muscle use and force for group A) | | | Neck  (Highest score=6) | | Trunk  (Highest score=6) | | Legs  (Highest score=2) | | Score B  (Neck, Trunk, Legs  Postures) | | Muscle Use  for Group B (Highest score=1) | | Force  for Group B (Highest score=3) | | Score D  (Score B+muscle use and force for group B) | | Grand Score  (Highest score=7) | | |
| --- | --- | --- | --- | --- | --- | --- | --- | --- | --- | --- | --- | --- | --- | --- | --- | --- | --- | --- | --- | --- | --- | --- | --- | --- | --- | --- | --- | --- | --- | --- | --- | --- | --- | --- | --- | --- |
|  | **Lt.** | **Rt.** | **Lt.** | **Rt.** | **Lt.** | **Rt.** | **Lt.** | **Rt.** | **Lt.** | **Rt.** | **Lt.** | **Rt.** | **Lt.** | | **Rt.** | **Lt.** | | **Rt.** |  | |  | |  | |  | |  | |  | |  | | **Lt.** | | **Rt.** |  |
| 01 | 2 | 2 | 2 | 2 | 3 | 3 | 1 | 1 | 3 | 3 | 1 | 1 | 2 | | 0 | 6 | | 4 | 3 | | 3 | | 2 | | 5 | | 1 | | 2 | | 8 | | 7 | | 6 |  |
| 02 | 1 | 1 | 2 | 2 | 3 | 4 | 1 | 1 | 3 | 3 | 1 | 1 | 0 | | 0 | 4 | | 4 | 3 | | 4 | | 2 | | 6 | | 1 | | 2 | | 9 | | 6 | | 6 |  |
| 03 | 1 | 2 | 2 | 2 | 4 | 3 | 1 | 1 | 3 | 3 | 1 | 1 | 0 | | 0 | 4 | | 4 | 4 | | 4 | | 2 | | 7 | | 1 | | 2 | | 10 | | 6 | | 6 |  |
| 04 | 1 | 2 | 2 | 2 | 2 | 2 | 1 | 1 | 3 | 2 | 1 | 1 | 0 | | 0 | 4 | | 3 | 3 | | 3 | | 2 | | 5 | | 1 | | 2 | | 8 | | 6 | | 6 |  |
| 05 | 1 | 1 | 2 | 2 | 3 | 4 | 1 | 1 | 3 | 3 | 1 | 1 | 0 | | 0 | 4 | | 4 | 3 | | 4 | | 2 | | 6 | | 1 | | 2 | | 9 | | 6 | | 6 |  |
| 06 | 1 | 1 | 2 | 2 | 4 | 4 | 1 | 1 | 3 | 3 | 1 | 1 | 0 | | 0 | 4 | | 4 | 4 | | 4 | | 2 | | 7 | | 1 | | 2 | | 10 | | 6 | | 6 |  |
| 07 | 1 | 1 | 2 | 2 | 2 | 2 | 1 | 1 | 2 | 2 | 1 | 1 | 0 | | 0 | 3 | | 3 | 4 | | 4 | | 1 | | 7 | | 1 | | 2 | | 9 | | 6 | | 6 |  |
| 08 | 1 | 1 | 2 | 2 | 2 | 3 | 1 | 1 | 3 | 2 | 1 | 1 | 0 | | 0 | 4 | | 3 | 3 | | 4 | | 1 | | 5 | | 1 | | 2 | | 7 | | 6 | | 6 |  |
| 09 | 1 | 1 | 2 | 2 | 4 | 4 | 2 | 2 | 3 | 3 | 1 | 1 | 0 | | 0 | 4 | | 4 | 4 | | 3 | | 1 | | 6 | | 1 | | 2 | | 8 | | 6 | | 6 |  |
| 10 | 2 | 1 | 2 | 2 | 4 | 4 | 2 | 2 | 3 | 4 | 1 | 1 | 0 | | 0 | 4 | | 5 | 3 | | 4 | | 2 | | 6 | | 1 | | 2 | | 9 | | 7 | | 6 |  |
| 11 | 1 | 1 | 2 | 2 | 3 | 3 | 1 | 1 | 3 | 3 | 1 | 1 | 0 | | 0 | 4 | | 4 | 4 | | 3 | | 1 | | 6 | | 1 | | 2 | | 8 | | 6 | | 6 |  |
| 12 | 1 | 2 | 2 | 2 | 3 | 4 | 1 | 1 | 4 | 3 | 1 | 1 | 0 | | 0 | 5 | | 4 | 4 | | 4 | | 2 | | 7 | | 1 | | 2 | | 10 | | 6 | | 7 |  |
| 13 | 1 | 1 | 2 | 2 | 3 | 4 | 1 | 1 | 3 | 3 | 1 | 1 | 0 | | 0 | 4 | | 4 | 4 | | 4 | | 2 | | 7 | | 1 | | 2 | | 10 | | 6 | | 6 |  |
| 14 | 1 | 1 | 2 | 2 | 4 | 4 | 2 | 2 | 3 | 3 | 1 | 1 | 0 | | 0 | 4 | | 4 | 5 | | 3 | | 2 | | 8 | | 1 | | 2 | | 11 | | 6 | | 6 |  |
| 15 | 1 | 2 | 2 | 2 | 3 | 2 | 1 | 1 | 3 | 3 | 1 | 1 | 0 | | 0 | 4 | | 4 | 5 | | 4 | | 2 | | 8 | | 1 | | 2 | | 11 | | 6 | | 6 |  |
| 16 | 2 | 1 | 2 | 2 | 3 | 2 | 1 | 1 | 2 | 3 | 1 | 1 | 2 | | 0 | 5 | | 4 | 5 | | 4 | | 2 | | 8 | | 1 | | 2 | | 11 | | 7 | | 6 |  |
| 17 | 2 | 1 | 2 | 2 | 2 | 3 | 1 | 1 | 3 | 3 | 1 | 1 | 0 | | 0 | 4 | | 4 | 4 | | 4 | | 2 | | 7 | | 1 | | 2 | | 10 | | 6 | | 6 |  |
| 18 | 1 | 2 | 2 | 2 | 2 | 4 | 1 | 1 | 4 | 2 | 1 | 1 | 0 | | 0 | 5 | | 3 | 4 | | 4 | | 1 | | 7 | | 1 | | 2 | | 9 | | 6 | | 7 |  |
| 19 | 1 | 2 | 2 | 2 | 3 | 3 | 1 | 1 | 3 | 3 | 1 | 1 | 0 | | 0 | 4 | | 4 | 4 | | 3 | | 1 | | 6 | | 1 | | 2 | | 8 | | 6 | | 6 |  |
| 20 | 2 | 1 | 2 | 2 | 4 | 4 | 1 | 1 | 3 | 4 | 1 | 1 | 0 | | 0 | 4 | | 5 | 3 | | 3 | | 2 | | 5 | | 1 | | 2 | | 7 | | 7 | | 6 |  |
| 21 | 1 | 2 | 2 | 2 | 3 | 3 | 1 | 1 | 3 | 3 | 1 | 1 | 0 | | 0 | 4 | | 4 | 4 | | 3 | | 2 | | 7 | | 1 | | 2 | | 10 | | 6 | | 6 |  |
| 22 | 1 | 1 | 2 | 2 | 3 | 3 | 1 | 1 | 3 | 3 | 1 | 1 | 0 | | 0 | 4 | | 4 | 4 | | 4 | | 2 | | 7 | | 1 | | 2 | | 10 | | 6 | | 6 |  |
| 23 | 1 | 2 | 2 | 2 | 4 | 3 | 2 | 1 | 3 | 3 | 1 | 1 | 0 | | 0 | 4 | | 4 | 4 | | 4 | | 1 | | 7 | | 1 | | 2 | | 9 | | 6 | | 6 |  |
| 24 | 2 | 2 | 2 | 2 | 1 | 3 | 1 | 1 | 3 | 3 | 1 | 1 | 2 | | 0 | 5 | | 4 | 3 | | 2 | | 2 | | 4 | | 1 | | 2 | | 7 | | 7 | | 6 |  |
| 25 | 2 | 2 | 2 | 2 | 4 | 3 | 2 | 1 | 3 | 4 | 1 | 1 | 0 | | 0 | 4 | | 5 | 4 | | 2 | | 2 | | 6 | | 1 | | 2 | | 9 | | 7 | | 6 |  |
| 26 | 1 | 1 | 2 | 2 | 1 | 4 | 1 | 1 | 3 | 2 | 1 | 1 | 0 | | 0 | 4 | | 3 | 4 | | 2 | | 2 | | 6 | | 1 | | 2 | | 9 | | 6 | | 6 |  |
| 27 | 1 | 1 | 2 | 2 | 3 | 3 | 1 | 1 | 3 | 3 | 1 | 1 | 0 | | 0 | 4 | | 4 | 2 | | 5 | | 2 | | 7 | | 1 | | 2 | | 10 | | 6 | | 6 |  |
| 28 | 1 | 1 | 2 | 2 | 4 | 3 | 2 | 1 | 3 | 3 | 1 | 1 | 2 | | 0 | 6 | | 4 | 3 | | 2 | | 1 | | 3 | | 1 | | 0 | | 4 | | 6 | | 4 |  |
| 29 | 2 | 1 | 1 | 1 | 3 | 4 | 1 | 1 | 3 | 3 | 1 | 1 | 0 | | 0 | 4 | | 4 | 4 | | 1 | | 2 | | 5 | | 1 | | 2 | | 8 | | 6 | | 6 |  |
| 30 | 1 | 1 | 2 | 2 | 4 | 3 | 1 | 1 | 3 | 3 | 1 | 1 | 0 | | 0 | 4 | | 4 | 4 | | 1 | | 1 | | 5 | | 1 | | 0 | | 6 | | 6 | | 6 |  |

Lt. = Left; Rt. = Right

150
