# Supplementary material for: Ergonomic risk assessment of smartphone users using the Rapid Upper Limb Assessment (RULA) tool
Source: PLoS One. 2018 Aug 30;13(8):e0203394. doi: 10.1371/journal.pone.0203394 (PMC6117073; doi:10.1371/journal.pone.0203394)
Supplement: S2 Table — (DOCX) [file pone.0203394.s002.docx]

**S2 Table. The musculoskeletal disorders of university student smartphone users.**

| ID | Body regions | | | | | | | | | |
| --- | --- | --- | --- | --- | --- | --- | --- | --- | --- | --- |
|  | **Neck** | **Shoulder** | **Elbow** | **Wrist and hand** | **Upper back** | **Lower back** | **Hip and thigh** | **Knee** | **Ankle and foot** |  |
| 01 | 2 | 1 | 1 | 2 | 4 | 1 | 1 | 1 | 1 |  |
| 02 | 2 | 2 | 4 | 2 | 4 | 1 | 1 | 1 | 1 |  |
| 03 | 2 | 4 | 1 | 3 | 4 | 1 | 2 | 1 | 1 |  |
| 04 | 2 | 3 | 1 | 1 | 1 | 1 | 1 | 1 | 2 |  |
| 05 | 2 | 2 | 1 | 1 | 4 | 1 | 1 | 1 | 1 |  |
| 06 | 2 | 2 | 1 | 2 | 4 | 1 | 1 | 2 | 2 |  |
| 07 | 2 | 4 | 1 | 1 | 4 | 1 | 1 | 1 | 1 |  |
| 08 | 2 | 4 | 1 | 4 | 4 | 1 | 1 | 1 | 1 |  |
| 09 | 2 | 3 | 1 | 4 | 4 | 1 | 1 | 1 | 1 |  |
| 10 | 2 | 4 | 1 | 2 | 4 | 2 | 2 | 2 | 1 |  |
| 11 | 2 | 1 | 1 | 1 | 1 | 1 | 1 | 1 | 1 |  |
| 12 | 2 | 4 | 1 | 1 | 3 | 1 | 1 | 1 | 1 |  |
| 13 | 2 | 3 | 1 | 1 | 4 | 1 | 1 | 1 | 1 |  |
| 14 | 2 | 2 | 1 | 2 | 4 | 1 | 1 | 2 | 1 |  |
| 15 | 2 | 4 | 2 | 2 | 4 | 2 | 2 | 2 | 1 |  |
| 16 | 2 | 1 | 1 | 1 | 2 | 1 | 1 | 1 | 1 |  |
| 17 | 2 | 2 | 1 | 1 | 4 | 1 | 1 | 1 | 2 |  |
| 18 | 2 | 1 | 1 | 1 | 3 | 1 | 1 | 1 | 1 |  |
| 18 | 2 | 2 | 1 | 2 | 1 | 2 | 1 | 1 | 1 |  |
| 20 | 2 | 1 | 1 | 1 | 1 | 1 | 2 | 1 | 1 |  |
| 21 | 2 | 4 | 1 | 4 | 4 | 2 | 1 | 1 | 1 |  |
| 22 | 2 | 4 | 1 | 1 | 1 | 1 | 1 | 1 | 1 |  |
| 23 | 2 | 1 | 1 | 1 | 4 | 2 | 1 | 1 | 1 |  |
| 24 | 1 | 2 | 1 | 1 | 1 | 1 | 1 | 1 | 1 |  |
| 25 | 2 | 4 | 1 | 1 | 1 | 2 | 1 | 1 | 1 |  |
| 26 | 1 | 1 | 1 | 1 | 1 | 1 | 1 | 1 | 1 |  |
| 27 | 2 | 4 | 1 | 1 | 1 | 1 | 1 | 1 | 1 |  |
| 28 | 1 | 1 | 1 | 1 | 1 | 2 | 1 | 1 | 1 |  |
| 29 | 2 | 2 | 1 | 1 | 4 | 2 | 1 | 1 | 1 |  |
| 30 | 2 | 4 | 1 | 1 | 1 | 2 | 1 | 1 | 1 |  |

Neck: 1 = no; 2 = yes,

Shoulder: 1 = no, 2 = only right side, 3 = only left side, 4 = both sides, Elbow: 1 = no, 2 = only right side, 3 = only left side, 4 = both sides,

Wrist and hand: 1 = no, 2 = only right side, 3 = only left side, 4 = both sides,

Upper back: 1 = no, 2 = only right side, 3 = only left side, 4 = both sides, Lower back: 1 = no; 2 = yes,

151

Hip and thigh: 1 = no; 2 = yes, Knee: 1 = no; 2 = yes, Ankle and foot: 1 = no; 2 = yes
